# A Phase Ib Study of the Simmitecan Single Agent and in Combination With 5-Fluorouracil/Leucovorin or Thalidomide in Patients With Advanced Solid Tumor

**DOI:** 10.3389/fphar.2022.833583

**Published:** 2022-07-22

**Authors:** Qi Zhang, Ting Deng, Fen Yang, Weijian Guo, Dan Liu, Jiajia Yuan, Changsong Qi, Yanshuo Cao, Qiuqiong Yu, Huiming Cai, Zhi Peng, Xicheng Wang, Jun Zhou, Ming Lu, Jifang Gong, Jian Li, Yi Ba, Lin Shen

**Affiliations:** ^1^ Department of Gastrointestinal Oncology, Key Laboratory of Carcinogenesis and Translational Research (Ministry of Education), Peking University Cancer Hospital and Institute, Beijing, China; ^2^ Tianjin Medical University Cancer Institute and Hospital, National Clinical Research Center for Cancer, Tianjin’s Clinical Research Center for Cancer, Key Laboratory of Cancer Prevention and Therapy, Tianjin, China; ^3^ Key Laboratory of Carcinogenesis and Translational Research (Ministry of Education), National Drug Clinical Trial Center, Peking University Cancer Hospital and Institute, Beijing, China; ^4^ Department of Medical Oncology, Fudan University Shanghai Cancer Center, Shanghai, China; ^5^ Haihe Biopharma Co., Ltd., Shanghai, China

**Keywords:** simmitecan, phase Ib study, solid tumor, 5-fluorouracil/leucovorin, thalidomide

## Abstract

**Background:** Simmitecan is a potent inhibitor of topoisomerase I with anti-tumor activity. This phase Ib trial was conducted to investigate the safety and anti-tumor effect of simmitecan alone or in combination with other drugs.

**Methods:** Eligible patients with advanced solid tumor had no further standard treatment options. Patients were allocated to receive simmitecan alone, simmitecan in combination with 5-fluorouracil (5-FU)/leucovorin (LV), or simmitecan in combination with thalidomide, 14 days a cycle, until disease progression or unacceptable toxicity occurred.

**Results:** A total of 41 patients were enrolled, with a median age of 55 (range 29–69) years. Among them, 13 patients received simmitecan monotherapy, 10 received simmitecan + 5-FU/LV, and 18 received simmitecan + thalidomide. No dose-limiting toxicity occurred. Overall, the most common grade 3/4 adverse event (AE) was neutropenia (46.2, 70.0, and 88.9%, respectively, in simmitecan, simmitecan + 5-FU/LV, and simmitecan + thalidomide cohorts), and treatment-related severe AEs included anemia and febrile neutropenia (7.7% each in simmitecan cohort), diarrhea (10% in simmitecan +5-FU/LV cohort), and febrile neutropenia (5.6% in simmitecan + thalidomide cohort). The majority of patients (24/41, 58.3%) had progressed on prior irinotecan; nevertheless, partial response was achieved in one colorectal cancer patients treated with simmitecan + thalidomide. The disease control rates of simmitecan, simmitecan + 5-FU/LV, and simmitecan + thalidomide cohorts were 46.2, 80.0, and 61.1%, respectively.

**Conclusion:** This study demonstrated a manageable safety profile of simmitecan as a single agent or as part of a combination therapy. There have not been any safety concerns with simmitecan in combination when compared to simmitecan alone. Simmitecan + 5-FU/LV regimen seemed to have a better efficacy. Nonetheless, the efficacy of this regimen needs to be further explored in the subsequent study.

**Clinical Trial Registration:** https://clinicaltrials.gov/, identifier NCT02870036

## Introduction

Camptothecin is a specific inhibitor of topoisomerase I (Topo I), and its derivatives irinotecan, topotecan, and hydroxycamptothecin have been widely used in the treatment of solid tumors. Simmitecan (active metabolite chimmitecan) is a novel 9-substituted lipophilic camptothecin. The inhibitory effect of chimmitecan on Topo I is stronger than that of SN38 (active metabolite of irinotecan) and topotecan (Huang et al., 2007). *In vitro*, chimmitecan has demonstrated a 2–3 times stronger cytotoxicity against tumor cells derived from 27 different origins than SN38, topotecan, and hydroxycamptothecin, and its anticancer activity against multidrug-resistant tumor cells is superior to that of topotecan and SN38. *In vivo*, chimmitecan has shown a significant inhibitory effect on tumor growth in mouse subcutaneous xenograft models established using human source pancreatic, colon, lung, and liver cancer cells (Huang et al., 2007).

Previous phase Ia results (NCT01832298) had provided safety data of the simmitecan single agent for patients with advanced solid tumor. A total of 39 patients were enrolled and treated at seven different dose levels [12.5, 25, 50, 80, 120, 160, and 180 mg/m^2^, every 2 weeks (Q2W)], and based on the results, the maximum tolerated dose (MTD) of simmitecan alone was determined to be 120 mg/m^2^ [totally seven patients were treated at this dose level, and no dose-limiting toxicity (DLT) events occurred] (unpublished data). The common adverse events (AEs) in the study were manageable hematological toxicity and gastrointestinal reactions.

Irinotecan plus 5-fluorouracil (5-FU)/leucovorin (LV) (FOLFIRI) has been demonstrated to be of benefit for patients with metastatic colorectal cancer (mCRC), where treatment options are still limited (Douillard et al., 2000). As mentioned above, chimmitecan is superior in anti-tumor effect to irinotecan in preclinical models; therefore, it is worth exploring the efficacy of the combination of simmitecan and 5-FU/LV (Huang et al., 2007).

Besides, several studies had indicated that thalidomide in combination with irinotecan could reduce severe diarrhoea induced by irinotecan (Govindarajan, 2000; Fadul et al., 2008). Thalidomide had been proposed to have several anti-tumor mechanisms, including antiangiogenic and immunomodulatory (Wang et al., 2016). Our preclinical data derived from mouse CT26 colon cancer xenografts had shown that the combination of chimmitecan and thalidomide provided a significant tumor growth inhibitory effect compared with chimmitecan alone (unpublished data).

The present phase Ib study was designed to investigate the safety, preliminary anti-tumor effects, and pharmacokinetics (PK) of simmitecan alone or in combination therapies.

## Patients and Methods

### Study Design

This phase Ib, multi-center, open-label study consisted of three separate treatment parts: simmitecan single agent (Part 1) and simmitecan in combination with 5-FU/LV (Part 2) or with thalidomide (Part 3). The study was registered on clinicaltrials.gov (NCT02870036).

The declared MTD of the simmitecan single agent was 120 mg/m^2^ Q2W, and the recommended dose for expansion was 80 mg/m^2^ Q2W based on the results of the phase Ia study. Therefore, simmitecan 50 mg/m^2^ Q2W (lower than this dose level may not guarantee patient benefits) was the selected starting dose (two dose level lower than the MTD of simmitecan monotherapy) in this single agent and combined dose escalation study. In addition, simmitecan 80 mg/m^2^ Q2W was expected to be the maximum dose in combination with 5-FU/LV or thalidomide in this study.

In the single agent study (Part 1), enrolled patients were planned to receive a 90-min infusion of 50, 80, or 120 mg/m^2^ of simmitecan Q2W (three patients at each dose level), and the cohort of 80 mg/m^2^ would be expanded.

Part 2 was a modified 3 + 3 dose escalation study. The starting dose of simmitecan was 50 mg/m^2^ Q2W, and the following dose of simmitecan was decided by Site Monitoring Committee (SMC) according to the established principles and obtained safety and efficacy data; 5-FU/LV was administrated as a fixed dose (LV 400 mg/m^2^, 5-FU bolus at 400 mg/m^2^ and 5-FU continuous infusion at 2,400 mg/m^2^, Q2W).

Part 3 was a traditional 3 + 3 dose escalation. Oral thalidomide was administrated at 50 or 100 mg/d. The combination of simmitecan and thalidomide is shown in Table 1. The cohort in which less than 1/6 patients experienced DLT and more than one patient achieved anti-tumor response [complete response (CR), partial response (PR), or stable disease (SD)] for 12 weeks after the baseline would be open to dose expansion by SMC.

**TABLE 1:** Combination of simmitecan and thalidomide.

Dose cohort	Simmitecan (mg/m^2^)	Thalidomide (mg)
0	50	50
1	65	50
2	80	50
3	65	100
4	80	100

DLT was defined as any of the following treatment-related toxicities during the first cycle of treatment: grade 4 neutropenia lasting 24 h or more, grade 3 neutropenia associated with fever, grade ≥3 neutropenia associated with grade 2 diarrhea, grade 4 thrombocytopenia, grade 3 thrombocytopenia with bleeding or grade 3 thrombocytopenia for more than 1 week, grade ≥3 non-hematologic toxicity (except alopecia and fatigue), grade 2 diarrhea lasting ≥7 days despite maximal supportive care (needing treatment with loperamide hydrochloride), and grade ≥3 nausea or vomiting despite maximal supportive care. Other clinically significant and/or unacceptable toxicities were evaluated by the SMC. The MTD was defined as the maximum dose at which the incidence of DLTs is less than 33.3% (1/3) in Part 2 and at less than 16.7% (1/6) in Part 3 during the DLT assessment window (within 14 days after administration of simmitecan). Treatment was continued until disease progression, unacceptable toxicity, or discontinuation at the patient’s request or death.

### Patients

Patients aged 18–70 years old with histologically or cytologically confirmed advanced solid tumors were recruited; only patients suitable for the treatment of simmitecan in combination with 5-FU/LV (determined by the investigator) were allowed entering Part 2, and only patients with gastrointestinal tumors were allowed into Part 3. Enrolled patients were refractory to standard treatment regimens or where no available standard therapy existed; all of them had evaluable lesions; all prior treatment-related toxicities had resolved to no greater than grade 1 before enrollment. Patients must have a good Eastern Cooperative Oncology Group (ECOG) performance status of 0 or 1; a life expectancy of 12 weeks or longer; and adequate bone marrow, liver, and renal functions. The key exclusion criteria were a history of administration of irinotecan in 3 months, cardiac disease with significant clinical symptoms, significant gastrointestinal abnormalities, active hepatitis, clinically serious infection, and uncontrolled brain metastases. Full inclusion and exclusion criteria were provided in the supplementary material.

The study protocol was approved by the independent ethics committee review board of each participant center. The study was conducted in accordance with the Declaration of Helsinki and Good Clinical Practice. All the patients were required to provide written informed consent before any study-related procedures were performed.

### Study Endpoints and Assessment

The primary endpoints of the study were to describe the DLT of simmitecan in combination with 5-FU/LV or thalidomide, respectively. The second endpoints included safety, efficacy, and the PK characteristics of simmitecan alone and in combination with 5-FU/LV or thalidomide.

AEs were assessed and graded according to the Common Terminology Criteria for Adverse Events (CTCAE) v4.03. Treatment emergent adverse events (TEAEs) were defined as AEs that occurred or worsened at or after the first dose of study treatment but no later than 32 days after the last dose. For each event, the highest degree of severity reached would be reported. The causal relationship between each AE and study treatment was classified as definitely irrelated, unlikely related, likely related, and definitely related. Dose modification would be permitted from cycle 2 according to the severity of the toxicities. The rules of dose de-escalation according to AEs are shown in Supplementary Table S1. When a patient experienced a grade 3/4 treatment-related AE, drug administration would be suspended until the AE resolved to baseline or grade 1, and the dose of resumption of treatment would be modified according to the principle in Supplementary Table S1.

Tumor measurements were performed using computed tomography or magnetic resonance imaging at baseline and every 6 weeks (±7 days) until progressive disease (PD), unacceptable toxicity, discontinuation at the patient’s request, or death. Tumor response was evaluated as per Response Evaluation Criteria in Solid Tumors (RECIST) v1.1. The objective response rate (ORR) (including CR and PR), disease control rate (DCR) (including CR, PR, and SD), overall survival (OS), and progression-free survival (PFS) were also evaluated.

### Pharmacokinetics Evaluation

Blood samples for pharmacokinetic analysis were collected in EDTA-K2 evacuated tubes at the following time points: pre-dose, 45 and 90 min after the start of infusion, and 0.5, 1, 2, 4, 8, 12, 24, and 48 h (Part 2 and 3) or 72 h (Part 1) after the end of infusion. The blood samples were subsequently centrifuged (3,500 rpm at 4 °C for 10 min), and obtained plasma samples were stored at −80 °C pending analysis.

The plasma concentrations of simmitecan and chimmitecan were determined using a validated liquid chromatography–tandem mass spectrometry (LC–MS/MS) system with irinotecan and SN38 used as the internal standard (IS) as previously described by our team (Zhou et al., 2021). The compounds were extracted using the protein precipitation method and detected as doubly charged ions. The multiple reaction monitor (MRM) transitions were m/z 599.3→m/z (124 + 345) for simmitecan, m/z 405→m/z (305 + 361) for chimmitecan, m/z 587→167 for irinotecan, and m/z 393→m/z (249 + 293) for SN38. Simmitecan and chimmitecan were identified and quantified over a theoretical concentration range of 1.0–500 ng/ml and 0.25–125 ng/ml, respectively.

The PK parameters were derived from plasma concentration–time data using the non-compartmental analysis (NCA) method from WinNonlin 6.3 (Pharsight Corp. Mountain View, CA, United States). The primary PK parameters included peak plasma concentration (C_max_), time to peak plasma concentration (T_max_), terminal phase half-life (t_1/2_), and the area under the plasma concentration–time curve from zero to the last time point (AUC_0−t_) and from zero to infinity (AUC_0-∞_).

### Statistical Analysis

The study was mainly based on descriptive statistical analysis. Different analysis subsets were adopted to evaluate the endpoints: the full analysis set (patients who received at least one dose of simmitecan), pharmacokinetic analysis set (patients who received at least one dose of simmitecan and their blood sample was collected and detected as planned), DLT analysis set (patients who had evaluable DLT in the first cycle), safety analysis set (patients who received at least one dose of simmitecan), and efficacy-evaluable analysis set (patients who received at least one dose of simmitecan and 5-FU/LV or thalidomide with at least one adequate tumor assessment both at baseline and after treatment). The Mann–Whitney test was adopted to compare PK parameters between the two groups using GraphPad Prism software v7.0. A *p*-value less than 0.05 was considered to be significant. The 90% confidence intervals were derived from the slope parameter (β), with a value of 1 indicative of 100% dose proportion.

## Results

### Patients’ Characteristics

**FIGURE 1:**
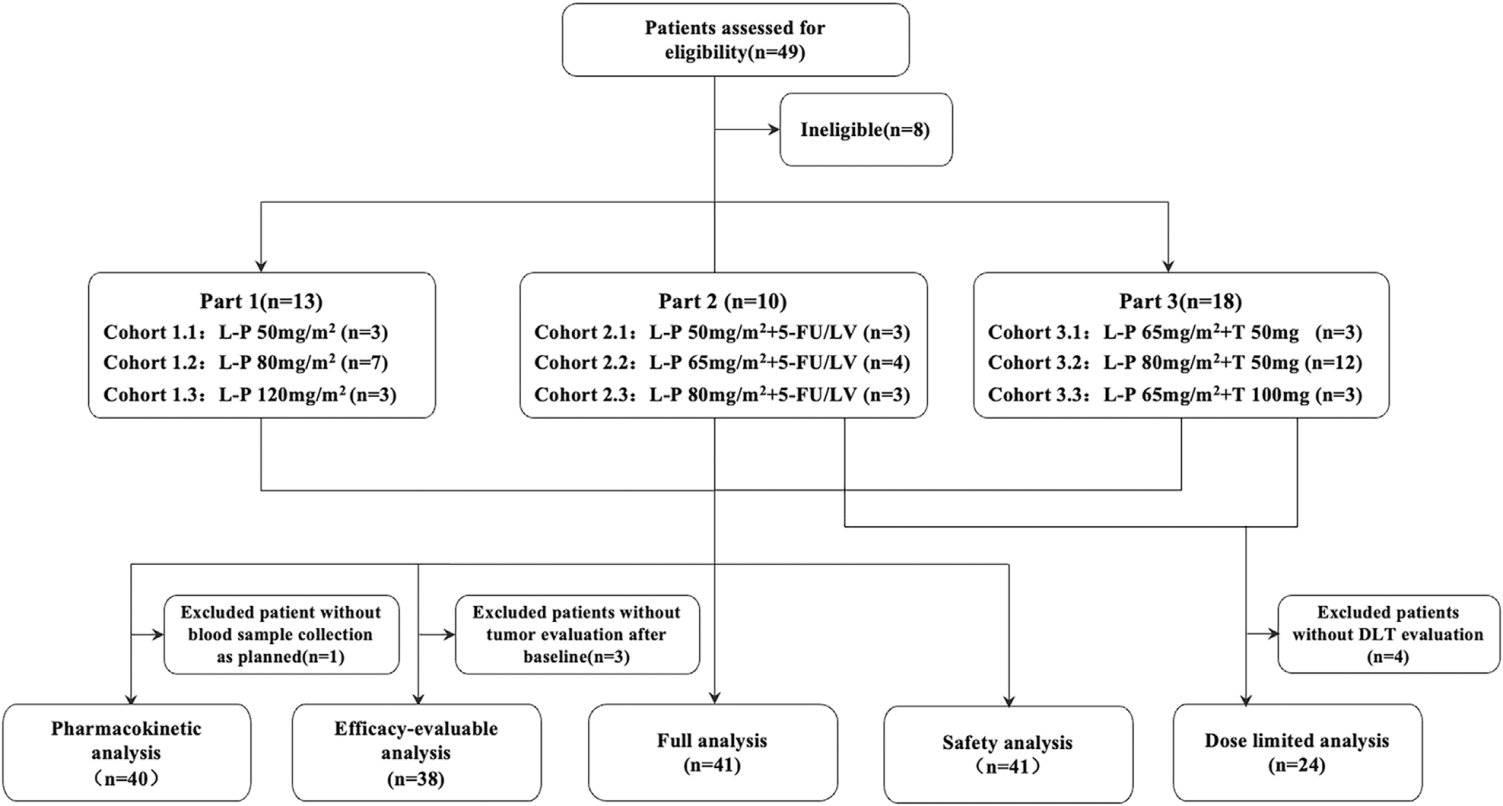
Study profile. The MTD of simmitecan single agent was 120 mg/m^2^ every 2 weeks (Q2W), and the recommended dose for expansion was 80 mg/m^2^ Q2W based on the results of phase Ia study. Simmitecan 50 mg/m^2^ Q2W was the selected starting dose in the single agent and combined dose escalation study, whereas simmitecan 80 mg/m^2^ Q2W was the expected highest dose in combination with 5-FU/LV or thalidomide in this study. In Part 2, the following dose of simmitecan was decided to be 65 mg/m^2^ or 80 mg/m^2^, with a fixed dose of 5-FU/LV (LV 400 mg/m^2^, 5-FU bolus at 400 mg/m^2^ and 5-FU continuous infusion at 2,400 mg/m^2^, Q2W), where there was a patient who should have received 80 mg/m^2^ of simmitecan and mistakenly received 65 mg/m^2^, and this case was not removed from the full analysis set after expert data review. In Part 3, the expansion–dose was determined to be simmitecan 80 mg/m^2^ in combination with thalidomide 50 mg. L-P, simmitecan; 5-FU/LV, 5-fluorouracil/leucovorin; T, thalidomide.

Between 10 October 2016 and 28 February 2019, 41 patients were enrolled. Among them, 13 patients entered Part 1 and received simmitecan monotherapy (three at 50 mg/m^2^, seven at 80 mg/m^2^, and three at 120 mg/m^2^), 10 patients entered Part 2 and received simmitecan (three at 50 mg/m^2^, four at 65 mg/m^2^, and three at 80 mg/m^2^) in combination with 5-FU/LV, and 18 patients entered Part 3 and received simmitecan in combination with thalidomide (three at simmitecan 65 mg/m^2^ + thalidomide 50 mg, 12 at simmitecan 80 mg/m^2^ + thalidomide 50 mg, and three at simmitecan 65 mg/m^2^ + thalidomide 100 mg) (Figure 1). In Part 2, there was a patient who should have received 80 mg/m^2^ of simmitecan and mistakenly received 65 mg/m^2^, and this case was not removed from the full analysis set after evaluation of historical data on expert reviews.

The baseline characteristics are shown in Table 2. The median age was 55 (range 29–69) years. The most prevalent cancer type was colorectal cancer (73.2%, *n* = 30). The median number of prior regimens for advance disease was 3. Also, 58.3% (*n* = 24) of patients had received irinotecan before enrollment. The database cutoff date was 27 September 2019, when all patients had finished the treatment and 17 patients had died because of disease progression. The median follow-up was 8.6 months (interquartile range 5.9–14.4).

**TABLE 2:** Baseline characteristics.

Demographics and disease characteristics	Patients (*n* = 41), *n*%
Median age, years (range)	55 (29–69)
Gender	
Male	20 (48.8)
Female	21 (51.2)
ECOG PS	
0	21 (51.2)
1	20 (48.8)
Cancer type	
Colorectal cancer	30 (73.2)
Breast cancer	2 (4.9)
Other gastrointestinal cancer^a^	9 (22.0)
Prior anticancer therapies	
Systemic	41 (100.0)
Radiotherapy	12 (29.3)
Surgery	35 (85.4)
Median prior chemotherapy regimens	3
Prior therapy with irinotecan	24 (58.3)

ECOG PS, Eastern Cooperative Oncology Group Performance Status.

a Other gastrointestinal cancers including one esophageal cancer, two gastric cancers, one small intestinal cancer, two pancreatic cancers, two cholangiocarcinomas, and two neuroendocrine carcinomas.

### Safety Assessments

No DLTs were observed, and the MTD was not reached. All patients experienced at least one TEAE (Table 3). The common TEAEs included leukopenia (92.3, 100.0, and 94.4% in Part 1, 2, and 3, respectively), neutropenia (53.8, 100.0, and 100.0% in Part 1, 2, and 3, respectively), and nausea (38.5, 70.0, and 72.2% in Part 1, 2, and 3, respectively). The most common grade 3/4 TEAE was neutropenia (46.2, 70.0, and 88.9% in Part 1, 2, and 3, respectively) (Table 4). Thirteen patients (31.7%) experienced any grade of diarrhea, and two of them (4.9%) (one at simmitecan 120 mg/m^2^ and one at simmitecan 80 mg/m^2^ + 5-FU/LV) experienced grade 3/4 diarrhea. No grade 5 AEs were observed.

**TABLE 3:** Summary of TEAEs (frequency >20% all grade).

Category	Part 1, *n* (%)				Part 2, *n* (%)				Part 3, *n* (%)			
	L-P 50 mg/m^2^(*n* = 3)	L-P 80 mg/m^2^(*n* = 7)	L-P 120 mg/m^2^ (*n* = 3)	Total (*n* = 13)	L-P 50 mg/m^2^+5-FU/LV (*n* = 3)	L-P 65 mg/m^2^+5-FU/LV (*n* = 4)	L-P 80 mg/m^2^+5-FU/LV (*n* = 3)	Total (*n* = 10)	L-P 65 mg/m^2^+T 50 mg (*n* = 3)	L-P-80 mg/m^2^+T 50 mg (*n* = 12)	L-P 65 mg/m^2^+T 100 mg (*n* =3)	Total (*n* = 18)
Any TEAE	3 (100.0)	7 (100.0)	3 (100.0)	13 (100.0)	3 (100.0)	4 (100.0)	3 (100.0)	10 (100.0)	3 (100.0)	12 (100.0)	3 (100.0)	18 (100.0)
Grade 3/4 TEAE	1 (33.3)	4 (57.1)	3 (100.0)	8 (61.5)	1 (33.3)	4 (100.0)	2 (66.7)	7 (70.0)	3 (100.0)	12 (100.0)	3 (100.0)	18 (100.0)
Nausea	1 (33.3)	3 (42.9)	1 (33.3)	5 (38.5)	2 (66.7)	3 (75.0)	2 (66.7)	7 (70.0)	3 (100)	9 (75.0)	1 (33.3)	13 (72.2)
Vomiting	1 (33.3)	2 (28.6)	1 (33.3)	4 (30.8)	2 (66.7)	2 (50.0)	3 (100.0)	7 (70.0)	2 (66.7)	7 (58.3)	0	9 (50.0)
Diarrhea	1 (33.3)	3 (42.9)	2 (66.7)	6 (46.2)	2 (66.7)	3 (75.0)	1 (33.3)	6 (60.0)	0	1 (8.3)	0	1 (5.6)
Constipation	0	2 (28.6)	0	2 (15.4)	0	1 (25.0)	0	1 (10.0)	1 (33.3)	2 (16.7)	0	3 (16.7)
Abdominal distension	2 (66.7)	1 (14.3)	0	3 (23.1)	0	0	0	0	0	0	0	0
Decreased appetite	0	2 (28.6)	3 (100.0)	5 (38.5)	0	0	1 (33.3)	1 (10.0)	2 (66.7)	1 (8.3)	0	3 (16.7)
Fever	0	1 (14.3)	1 (33.3)	2 (15.4)	1 (33.3)	1 (25.0)	1 (33.3)	3 (30.0)	0	2 (16.7)	0	2 (11.1)
Alopecia	0	3 (42.9)	2 (66.7)	5 (38.5)	3 (100.0)	2 (50.0)	1 (33.3)	6 (60.0)	1 (33.3)	4 (33.3)	2 (66.7)	7 (38.9)
Fatigue	0	2 (28.6)	1 (33.3)	3 (23.1)	1 (33.3)	2 (50.0)	1 (33.3)	4 (40.0)	2 (66.7)	2 (16.7)	1 (33.3)	5 (27.8)
Malaise	1 (33.3)	1 (14.3)	0	2 (15.4)	0	1 (25.0)	1 (33.3)	2 (20.0)	0	4 (33.3)	0	4 (22.2)
Dizziness	0	0	0	0	0	1 (25.0)	0	1 (10.0)	0	4 (33.3)	0	4 (22.2)
Leukopenia	2 (66.7)	7 (100)	3 (100)	12 (92.3)	3 (100)	4 (100.0)	3 (100)	10 (100.0)	3 (100.0)	11 (91.7)	3 (100)	17 (94.4)
Neutropenia	2 (66.7)	3 (42.9)	2 (66.7)	7 (53.8)	3 (100)	4 (100)	3 (100)	10 (100.0)	3 (100)	11 (91.7)	3 (100)	18 (100.0)
Anemia	0	1 (14.3)	2 (66.7)	3 (23.1)	0	0	2 (66.7)	2 (20)	3 (100)	11 (91.7)	3 (100)	17 (94.4)
Thrombocytopenia	0	0	1 (33.3)	1 (7.7)	1 (33.3)	2 (50.0)	3 (100.0)	6 (60.0)	1 (33.3)	4 (33.3)	1 (33.3)	6 (33.3)
Increased in ALT	0	1 (14.3)	1 (33.3)	2 (15.4)	2 (66.7)	0	1 (33.3)	3 (30.0)	3 (100)	5 (41.6)	0	8 (44.4)
Increased in AST	0	2 (28.6)	1 (33.3)	3 (23.1)		2 (50.0)	1 (33.3)	3 (30.0)	2 (66.7)	3 (25.0)	0	5 (27.8)
Increased in TBIL	0	0	1 (33.3)	1 (7.7)	2 (66.7)	1 (25.0)	0	3 (30.0)	1 (33.3)	4 (33.3)	1 (33.3)	6 (33.3)
Hyperbilirubinemia	0	0	2 (66.7)	2 (15.4)	0	0	0	0	0	0	0	0
Hypoproteinemia	0	1 (14.3)	1 (33.3)	2 (15.4)	0	0	0	0	1 (33.3)	0	0	1 (5.6)
Hematuria	1 (33.3)	1 (14.3)	0	2 (15.4)	2 (66.7)	0	0	2 (20.0)	0	0	0	0
Proteinuria	1 (33.3)	1 (14.3)	0	2 (15.4)	1 (33.3)	0	1 (33.3)	2 (20.0)	1 (33.3)	4 (33.3)	2 (66.7)	7 (38.9)

L-P, simmitecan; 5-FU/LV, 5-fluorouracil/leucovorin; T, thalidomide; TEAE, treatment emergent adverse event; ALT, alanine aminotransferase; AST, aspartate aminotransferase; TBIL, total bilirubin.

**TABLE 4:** Grade 3/4 TEAE.

Preferred term	Part 1, *n* (%)				Part 2, *n* (%)				Part 3, *n* (%)			
	L-P 50 mg/m^2^ (*n* = 3)	L-P 80 mg/m^2^ (*n* = 7)	L-P 120 mg/m^2^ (*n* = 3)	Total (*n* = 13)	L-P 50 mg/m^2^+5-FU/LV (*n* = 3)	L-P 65 mg/m^2^+5-FU/LV (*n* = 4)	L-P 80 mg/m^2^+5-FU/LV (*n* = 3)	Total (*n* = 10)	L-P 65 mg/m^2^+T 50 mg (*n* = 3)	L-P-80 mg/m^2^+T 50 mg (*n* = 12)	L-P 65 mg/m^2^+T 100 mg (*n* = 3)	Total (*n* = 18)
Vomiting	0	1 (14.3)	0	0	0	0	0	0	0	0	0	0
Diarrhea	0	0	1 (33.3)	1 (7.7)	0	0	1 (33.3)	1 (10.0)	0	0	0	0
Fatigue	0	0	0	0	0	1 (25.0)	0	1 (10.0)	0	0	0	0
Malaise	0	0	0	0	0	0	0	0	0	3 (25.0)	0	3 (16.7)
Decreased appetite	1 (33.3)	0	0	1 (7.7)	0	0	0	0	0	0	0	0
Abdominal pain	0	0	0	0	0	1 (25.0)	0	1 (10.0)	0	0	0	0
Bowel obstruction	0	0	0	0	0	1 (25.0)	0	0	0	0	1 (33.3)	1 (5.6)
Pulmonary embolism	0	0	0	0	0	0	0	0	0	1 (8.3)	0	1 (5.6)
Alopecia	0	0	0	0	0	0	0	0	0	0	1 (33.3)	1 (5.6)
Leukopenia	0	2 (28.6)	3 (100)	5 (38.5)	1 (33.3)	2 (50.0)	1 (33.3)	4 (40.0)	2 (66.7)	6 (50.0)	2 (66.7)	10 (55.5)
Neutropenia	0	4 (57.1)	2 (66.7)	6 (46.2)	1 (33.3)	4 (100)	2 (66.7)	7 (70.0)	2 (66.7)	11 (100.0)	3 (100)	16 (88.9)
Anemia	0	0	1 (33.3)	1 (7.7)	0	0	1 (33.3)	1 (10.0)	1 (33.3)	1 (8.3)	1 (33.3)	3 (16.7)
Thrombocytopenia	0	0	1 (33.3)	1 (7.7)	0	0	0	0	1 (33.3)	0	1 (33.3)	2 (11.1)
Increased in ALT	0	0	0	0	0	0	0	0	0	1 (8.3)	0	1 (5.6)
Increased in IBIL	0	0	0	0	0	0	0	0	0	1 (8.3)	0	0
Hypokalemia	0	0	1 (33.3)	1 (7.7)	0	0	0	0	0	0	0	0
Febrile Neutropenia	0	0	1 (33.3)	1 (7.7)	0	0	0	0	1 (33.3)	0	0	1 (5.6)

L-P, simmitecan; 5-FU/LV, 5-fluorouracil/leucovorin; T, thalidomide; TEAE, treatment emergent adverse event; ALT, alanine aminotransferase; IBIL, indirect bilirubin.

Nine patients (22.0%) experienced treatment emergent serious AEs (TESAEs), including three patients in Part 1 (one suffered anorexia at simmitecan 50 mg/m^2^, one febrile neutropenia, and one anemia at simmitecan 80 mg/m^2^), two in Part 2 (one suffered bowel obstruction at simmitecan 65 mg/m^2^ + 5-FU/LV, one diarrhea at simmitecan 80 mg/m^2^ + 5-FU/LV), and four in Part 3 (one suffered febrile neutropenia at simmitecan 65 mg/m^2^ + thalidomide 50 mg, one alkaline phosphatase elevation, one pulmonary embolism at simmitecan 80 mg/m^2^ + thalidomide 50 mg, and one bowel obstruction at simmitecan 65 mg/m^2^ + thalidomide 100 mg). Except bowel obstruction, the other TESAEs were considered to be related to treatments.

The median dose intensity for simmitecan was 97.8, 88.4, and 90.0% in Part 1, 2, and 3, respectively. The most frequent AE leading to dose modification was neutropenia. Dose modification of the study drug due to TEAEs is shown in Supplementary Table S2. One patient (10%) in Part 2 required dose reduction for treatment-related TEAE. Interruption of drug administration owing to treatment-related TEAEs occurred in 38.5, 40.0, and 55.6% of patients in Part 1, 2, and 3, respectively. Besides, 7.7% of patients in Part 1 and 16.7% of patients in Part 3 required discontinuation for treatment-related TEAE.

### Efficacy Assessments

**FIGURE 2:**
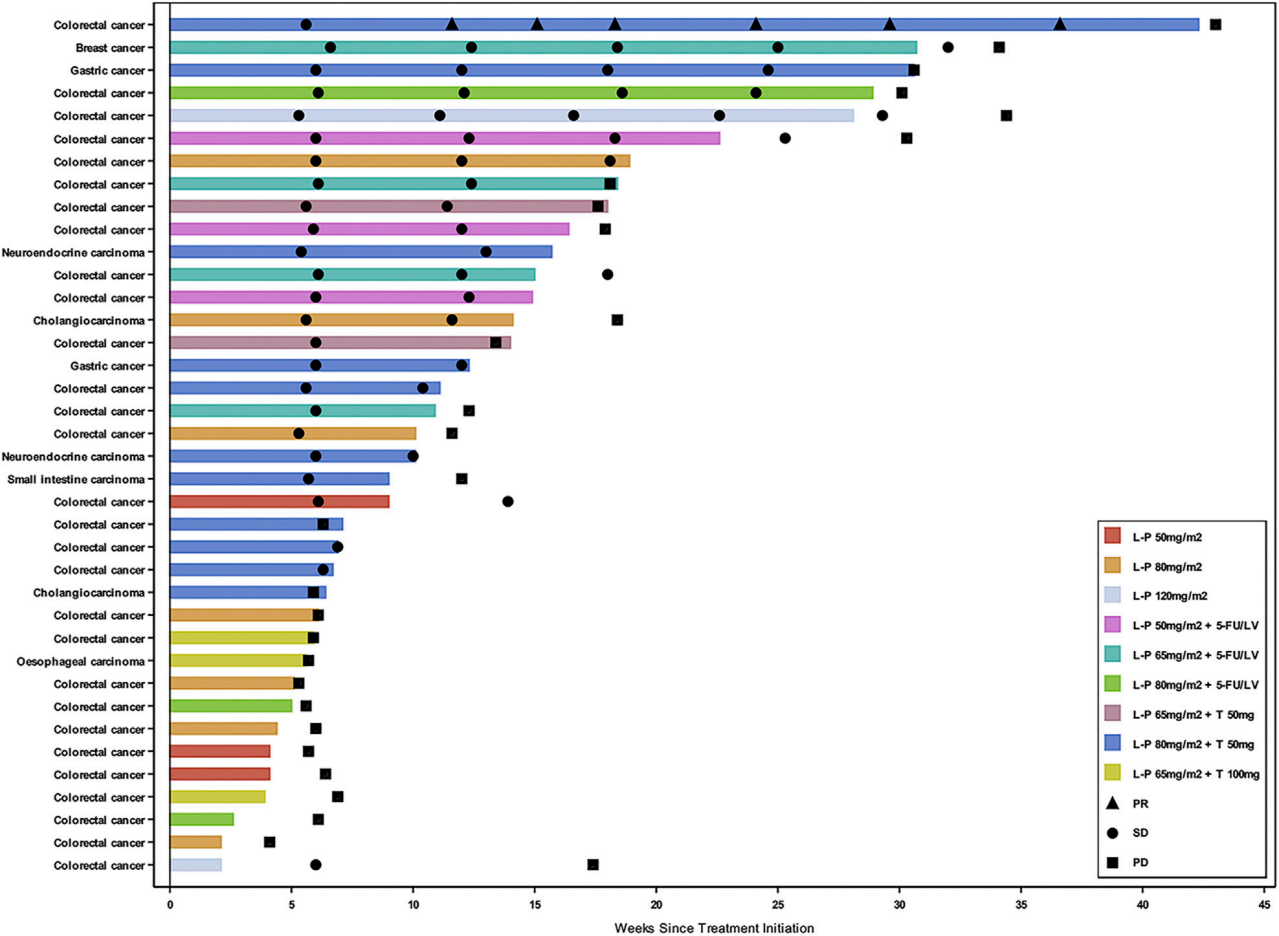
Swimmer plot by cancer type and dose level. Three patients who had clinical deterioration before an initial response assessment were excluded from the swimmer plot. Response assessment was performed in according to the Response Evaluation Criteria in Solid Tumors version 1.1. L-P, simmitecan; 5-FU/LV, 5-fluorouracil/leucovorin; T, thalidomide; PR, partial response; SD, stable disease; PD, progressive disease.

Thirty-eight patients (92.7%) who received study drug treatment were evaluable for efficacy. The median duration of treatment was 10.1 weeks (range 2.1–42.3) (Figure 2). The confirmed DCR was 46.2, 80.0, and 61.1% in Part 1, 2, and 3, respectively (Supplementary Table S3). A 44-year-old male with mCRC at simmitecan 80 mg/m^2^ + thalidomide 50 mg achieved a confirmed PR with a duration of 7.4 months.

In Part 1, the median PFS and median OS were 2.1 and 7.8 months, respectively. In Part 2, with the increasing dose level of simmitecan (50 mg/m^2^, 65 mg/m^2^, and 80 mg/m^2^), there was a downward trend of median PFS (5.5, 4.2, and 1.1 months), and the median OSs were 15.8, 15.8, and 13.7 months, respectively. In Part 3, the median PFSs were 3.1, 4.9, and 1.4 months at simmitecan 65 mg/m^2^ + thalidomide 50 mg, simmitecan 80 mg/m^2^ + thalidomide 50 mg, and simmitecan 65 mg/m^2^ + thalidomide 100 mg, respectively. As of the database cutoff date, no death occurred at simmitecan 80 mg/m^2^ + thalidomide 50 mg, and the median OS of this cohort was not yet reached; the median OS of patients was 8.3 and 5.1 months at simmitecan 65 mg/m^2^ + thalidomide 50 mg and simmitecan 65 mg/m^2^ + thalidomide 100 mg, respectively.

### Pharmacokinetics Analysis

**FIGURE 3:**
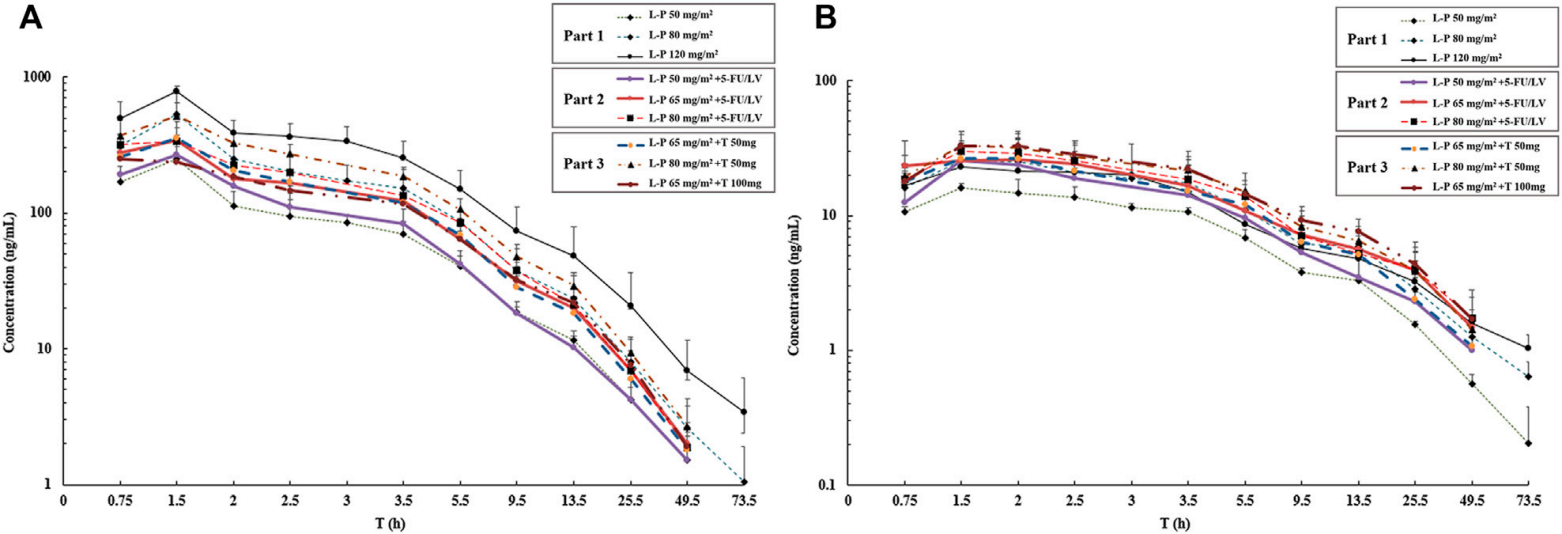
Mean logarithmic concentration vs time plot of simmitecan **(A)** and chimmitecan **(B)** following infusion of simmitecan as a single agent (Part 1) and in combination with 5-fluorouracil/leucovorin (Part 2) or thalidomide (Part 3). L-P, simmitecan; 5-FU/LV, 5-fluorouracil/leucovorin; T, thalidomide.

One patient without blood sample collection as planned in Part 1 was removed from the PK analysis. The mean plasma concentrations of simmitecan and chimmitecan-time profiles of the patients are presented in Figure 3. The corresponding PK parameters were calculated from these data and are listed in Table 5. Both simmitecan and chimmitecan reached C_max_ almost at the end of the simmitecan infusion. After that, a slow distribution phase and a terminal elimination phase were observed. There was a correspondingly positive correlation between systemic exposure of the study drug and the dose escalation across the whole study from 50 to 120 mg/m^2^ in Part 1 (mean C_max_ of 251–787 ng/ml, 16.6–26.4 ng/ml and mean AUC_0-∞_ of 933–3,530 h*ng/ml, 164–328 h*ng/ml for simmitecan and chimmitecan, respectively), from 50 to 80 mg/m^2^ in Part 2 (mean C_max_ of 268–396 ng/ml, 25.4–29.7 ng/ml and mean AUC_0-∞_ of 1,010–1,650 h*ng/ml, 246–356 h*ng/ml for simmitecan and chimmitecan, respectively), and from 65 to 80 mg/m^2^ in Part 3 (mean C_max_ of 257–517 ng/ml, 28.4–33.8 ng/ml and mean AUC_0-∞_ of 1,400–2,230 h*ng/ml, 266–387 h*ng/ml for simmitecan and chimmitecan, respectively). The slopes (β value) were 1.3 for C_max_ and 1.5 for AUC_0–t_ from linearity analysis of simmitecan in Part 1. The observed mean value of t_1/2_ was comparable between Part 1 and 2 for simmitecan (14.3 h vs.11.4 h, *p* = 0.11) and chimmitecan (24.3 h vs.20.9 h, *p* = 0.29). Shortened t_1/2_ values of simmitecan (10.3 h, *p* = 0.01) and chimmitecan (16 h, *p* = 0.048) were observed in Part 3 compared to those in Part 1. After administration of simmitecan in combination with 5-FU/LV or thalidomide, the mean C_max_ ratios of chimmitecan/simmitecan were significantly increased (8.2 and 7.4 for Part 2 and 3, respectively), as well as AUC_0–t_ ratios of chimmitecan/simmitecan (22.0) in Part 2 compared with those (the mean C_max_ and AUC_0–t_ ratio were 4.8 and 13.2, respectively) in Part 1 (*p* < 0.05), whereas the mean AUC_0–t_ ratios of the two analytes (18) were almost unchanged between Part 1 and 3 (*p* = 0.74).

**TABLE 5:** Pharmacokinetic parameters of simmitecan and chimmitecan.

PK Parameters		Dose Level (mg/m^2^)	^a^t_1/2_ (h)	^b^Tmax (h)	^a^Cmax (ng/ml)	^a^AUC_0-t_ (h*ng/ml	^b^AUC _0-∞_ (h*ng/ml)	^c^Cmax ratio (%)	^c^AUC_0-t_ ratio (%)
Simmitecan	Part 1	L-P 50 mg/m^2^ (n = 3)	12.0 (2.8)	1.53 (1.50, 1.53)	251 (59.4)	906 (117)	933 (104)	6.6	17.1
		L-P 80 mg/m^2^ (n = 6)^d^	13.5 (4.8)	1.53 (1.42, 1.63)	535 (224)	1890 (691)	1920 (701)	4.9	14.6
		L-P 120 mg/m^2^ (n = 3)	17.3 (1.8)	1.52 (1.48, 1.52)	787 (65.4)	3,450 (1,380)	3,530 (1,430)	3.0	7.9
	Part 2	L-P 50 mg/m^2^ + 5-FU/LV (n = 3)	13.4 (1.9)	1.50 (1.50, 1.52)	268 (53.0)	977 (149)	1,010 (146)	9.4	24.3
		L-P 65 mg/m^2^ + 5-FU/LV (n = 4)	10.7 (1.9)	1.50 (1.48, 1.55)	396 (89.6)	1,400 (238)	1,430 (238)	7.0	22.0
		L-P 80 mg/m^2^ + 5-FU/LV (n = 3)	10.3 (1.1)	1.50 (0.75, 1.50)	351 (86.3)	1,630 (113)	1,650 (117)	8.5	19.8
	Part 3	L-P 65 mg/m^2^ + T 50 mg (n = 3)	10.6 (1.0)	1.50 (1.50, 1.50)	357 (16.3)	1,420 (289)	1,450 (299)	7.9	18.3
		L-P 80 mg/m^2^ + T 50 mg (n = 12)	10.1 (1.0)	1.51 (1.47, 1.62)	517 (131)	2,190 (370)	2,230 (381)	5.9	16.1
		L-P 65 mg/m^2^ + T 100 mg (n = 3)	9.97 (1.0)	1.50 (0.78, 1.62)	257 (22.0)	1,370 (102)	1,400 (115)	13.1	27.6
Chimmitecan	Part 1	L-P 50 mg/m^2^ (n = 3)	16.8 (2.9)	1.53 (1.50, 2.02)	16.6 (1.60)	155 (14.3)	164 (13.9)	NA	NA
		L-P 80 mg/m^2^ (n = 6)^d^	21.2 (3.5)	2.05 (1.42, 3.57)	26.4 (8.10)	275 (52.6)	295 (56.0)		
		L-P 120 mg/m^2^ (n = 3)	34.8 (25.2)	1.52 (1.48, 2.50)	24.0 (17.2)	272 (155)	328 (114)		
	Part 2	L-P 50 mg/m^2^ + 5-FU/LV (n = 3)	25.0 (15.8)	1.50 (1.50, 1.52)	25.4 (16.8)	207 (85.8)	246 (63.3)	NA	NA
		L-P 65 mg/m^2^ + 5-FU/LV (n = 4)	17.8 (1.4)	1.55 (1.48, 1.98)	27.7 (12.0)	278 (58.4)	315 (59.0)		
		L-P 80 mg/m^2^ + 5-FU/LV (n = 3)	20.8 (6.5)	1.50 (1.50, 1.50)	29.7 (4.90)	298 (82.8)	356 (135)		
	Part 3	L-P 65 mg/m^2^ + T 50 mg (n = 3)	16.0 (4.6)	2.00 (1.50, 2.00)	28.4 (4.46)	240 (11.7)	266 (5.86)	NA	NA
		L-P 80 mg/m^2^ + T 50 mg (n = 12)	17.0 (3.7)	1.58 (1.47, 2.07)	33.8 (9.92)	321 (92.9)	358 (104)		
		L-P 65 mg/m^2^ + T 100 mg (n = 3)	16.0 (5.0)	2.00 (1.60, 2.62)	33.6 (4.05)	342 (86.6)	387 (124)		

a The data are shown as mean (SD).

b T_max_ is shown as median (minimum, maximum).

c Ratio = chimmitecan/simmitecan.

d One patient without blood sample collection as planned was removed from the PK analysis.

PK, pharmacokinetic; t_1/2_, terminal phase half-life; T_max_, time to peak plasma concentration; C_max_, peak plasma concentration; AUC_0-t_, the area under the plasma concentration–time curve from zero to the last time point; AUC_0-∞_, AUC, from time zero to infinity; L-P, simmitecan; 5-FU/LV, 5-fluorouracil/leucovorin; T, thalidomide; NA, not applicable.

## Discussion

This phase Ib study provided the safety profile, preliminary efficacy, and PK of simmitecan as a single agent and in combination with 5-FU/LV or thalidomide in patients with pretreated solid tumor. No DLT was observed during the study period.

Myelosuppression and gastrointestinal reaction, including nausea and vomiting, were the major TEAEs with simmitecan monotherapy. The toxicity profile of simmitecan in combination of 5-FU/LV, with respect to the types of AEs, seemed to be comparable with that of FOLFIRI (Douillard et al., 2000; Colucci et al., 2005), while the difference was less grade 3/4 diarrhea (10.0 versus 44.4%) and more all grade myelosuppression (100.0 versus 28.8%) observed in our study. What is more, with dose modification and symptomatic treatment, the results demonstrated a manageable toxicity profile, with no unexpected safety concerns.

Of note, only one patient receiving simmitecan in combination with thalidomide experienced grade 1/2 diarrhea, and no severe diarrhea was observed, which could occur in 40% of patients receiving irinotecan treatment (Douillard et al., 2000). *In vivo* and *in vitro* models demonstrated that coadministered thalidomide significantly attenuated diarrhea and intestinal histological lesions caused by irinotecan; the accompanied inhibition of tumor necrosis factor-alpha, interleukins 1 and 6 and interferon-gamma, and intestinal epithelial apoptosis suggested a possible mechanism by which thalidomide counteracted the diarrhea resulting from irinotecan (Yang et al., 2006). In a phase II study of thalidomide (400 mg/d)/irinotecan (300–350 mg/m^2^, every 3 weeks), out of 15 mCRC patients, only one patient suffered diarrhea after discontinuation of thalidomide because of skin rash and required hospitalization (Govindarajan et al., 2000). Besides, among patients with non-small-cell lung cancer, a previous study reported that the occurrence of grade 3/4 diarrhea was 7.0% after treatment with irinotecan, carboplatin, and thalidomide (Miller et al., 2006), whereas it was 16.3% (24/147) with irinotecan and carboplatin in another study (Ohe et al., 2007). All these results provided clinical evidence for protective effects of thalidomide against chemotherapy-induced gastrointestinal toxicity.

The anti-tumor activity of simmitecan combined with 5-FU/LV was noted in these heavily pretreated patients (90% were patients with mCRC), half of whom had received prior treatment with irinotecan. Although none of the patients on this regimen achieved PR, which might be partly due to the sample size, preliminary efficacy results indicated that 80% of patients achieved disease control. For reference, FOLFIRI as a third-line therapy for patients with mCRC resulted in an ORR of 6% and a DCR of 61% (André et al., 1999). Additionally, phase III studies showed that regorafenib and fruquintinib, which have been approved for use in refractory mCRC patients in China, induced DCRs of 51% (CUNCUR) and 62.2% (FRESCO), respectively (Li et al., 2015; Li et al., 2018). It was difficult to determine the recommended phase II dose due to the absence of DLT. However, in our study, simmitecan at a dose level of 50 mg/m^2^ or 65 mg/m^2^ was more promising in efficacy than that at 80 mg/m^2^, when combined with 5-FU/LV, whereas the dose level of 50 mg/m^2^ resulted in fewer grade 3/4 TEAEs (33.3%) than the dose level of 65 mg/m^2^ (100.0%). Therefore, simmitecan 50 mg/m^2^ combined with 5-FU/LV might be a more appropriate treatment option.

In our study, one CRC patient achieved PR (ORR 8.3%) after treatment with simmitecan (at 80 mg/m^2^) in combination with 50 mg thalidomide, and the DCR at this dose level was 75.0%. As mentioned above, in the phase II study of second-line treatment with thalidomide/irinotecan in mCRC patients, preliminary results showed that the ORR was 28.6% (4/14) and the DCR was 71.4% (10/14) (Govindarajan, 2002). Although this study provided preliminary efficacy and safety results of thalidomide/irinotecan, there were no subsequent reports on this regimen in the treatment of mCRC, which might have arisen from a lack of efficacy after increasing the sample size or other safety concerns leading to study failure. Therefore, further studies are needed to confirm it.

One of the objectives of the study was to characterize the PK and explore the exposure–response relationship of simmitecan after administration of simmitecan as single agent or in combination with 5FU/LV or thalidomide. The PK results showed that the increasing rate of exposure of simmitecan was higher than that of dose escalation from 50 to 120 mg/m^2^ due to the β value larger than 1. Thalidomide might accelerate the elimination of simmitecan and chimmitecan from the body and results in a decreased mean t_1/2_ value (*p* < 0.05) by inducing the esterase and CYP3A because simmitecan was the substrate of hepatic esterase and CYP3A and chimmitecan was the substrate of CYP3A. The primary PK parameters of simmitecan and chimmitecan in all three parts demonstrated that a higher metabolic rate from simmitecan (prodrug) to chimmitecan (active metabolite) was achieved after administration of simmitecan in combination with 5-FU/LV, evidenced by the significantly elevated ratios of exposure (*p* < 0.05), which was consistent with the clinical result of higher DCR (80.0%).

The major limitations of this study were a small sample size and a non-randomized controlled study. In addition, owing to the study design, most of the patients had been heavily treated and some of them had been exposed to irinotecan (58.3%). It is unclear whether irinotecan resistance could affect the efficacy of simmitecan, although the anti-tumor effect of simmitecan has already been observed in irinotecan-treated patients.

In all, this study showed that the safety profiles of simmitecan were manageable either as a single agent or in combination with 5-FU/LV or thalidomide. Simmitecan in combination with 5-FU/LV was more promising in efficacy than simmitecan alone. Nonetheless, the efficacy of this regimen should be further explored in the subsequent study.

## Data Availability

The data that support the findings of our study are available on request from the corresponding author. The data are not publicly available due to privacy or ethical restrictions.
