# Supplementary material for: A Phase Ib Study of the Simmitecan Single Agent and in Combination With 5-Fluorouracil/Leucovorin or Thalidomide in Patients With Advanced Solid Tumor
Source: Front Pharmacol. 2022 Jul 22;13:833583. doi: 10.3389/fphar.2022.833583 (PMC9355729; doi:10.3389/fphar.2022.833583)
Supplement: Supplementary file 1 [file DataSheet1.pdf]

Supplementary Material

1 Patients

Inclusion criteria:

1) Patients who have fully understood the study, and are willing to sign the informed consent (ICF);
2) Patients with advanced solid tumor confirmed by histopathology and/or cytology were enrolled in Parts 1 and 2 of the study; In Part 3 of the study, patients with advanced gastrointestinal tumor (including biliary tumor [gallbladder carcinoma, intra- or extra-hepatic biliary carcinoma], pancreatic carcinoma, hepatocellular carcinoma, esophageal carcinoma, gastric carcinoma, colorectal carcinoma, gastrointestinal stromal carcinoma, and gastro-entero-pancreatic neuroendocrine carcinoma) confirmed by histopathology were enrolled; Patients being enrolled in part 2 of the study should be those with advanced solid tumor who are suitable for therapeutic regimen of simmitecan combined with 5 FU/LV as judged by the investigator;
3) Patients who had failed standard treatments for advanced cancer, or are intolerant to the current standard treatments, or no suitable standard treatments available for advanced cancer;
4) Patients who have at least one measurable lesion (according to RECIST, version 1.1); note: the lesions previously treated with radiotherapy cannot be regarded as the target lesion, unless the lesion showed clear progression after radiotherapy;
5) Patients with Eastern Cooperative Oncology Group (ECOG) performance status score of 0 or 1;
6) Age≥ 18 years and ≤ 70 years, male or female is allowed;
7) Patients with life expectancy of 12 weeks or more;
8) Patients with appropriate organ function as documented by:
    a) absolute neutrophil count ≥ 1.5 × 109/L;
    b) hemoglobin ≥ 9 g/dL (without RBC transfusion within 14 days);
    c) platelet count ≥ 100 × 109/L.
    d) serum total bilirubin ≤1.5 times of upper limit of normal (ULN) (for the patients with Gilbert syndrome, total bilirubin is allowed to be ≤ 3 × ULN and direct bilirubin is allowed to be 1.5 × ULN);
    e) aspertate aminotransferase (AST), alanine aminotransferase (ALT) ≤ 2.5 × ULN, or AST and ALT ≤ 5 × ULN for patients with liver metastasis;
    f) creatinine clearance ≥ 50 mL/min (calculated by MDRD equation, see appendix 7);
    g) international normalized ratio (INR) ≤1.5 × ULN, or activated partial thromboplastin time (aPTT) ≤ 1.5 × ULN (INR only for patients who were not given anticoagulant therapy).
9) Patients with negative hepatitis b surface antigen; for patients with positive hepatitis b surface antigen, quantitative result of hepatitis b virus (HBV) DNA should be lower than 1000 cps/mL;

10) Toxicity events caused by previous treatments, surgery or radiotherapy have recovered to CTCAE grade 0 or 1 (except for alopecia);
11) Female patients are eligible for the study if the following conditions are met:
    a) Patients have no fertility (i.e., physiological infertility), including postmenopausal period (complete menolipsis for more than 1 year) or documented irreversible sterilization operation including hysterectomy, bilateral ovariectomy, or bilateral salpingectomy (instead of tubal ligation);
    b) For patients of childbearing potential, the results of serum pregnancy test screening should be negative (within 7 days before the first dose of investigational drug), and breastfeeding is not allowed before the start of the study and throughout the study. Moreover, the patients should agree to take effective contraception measures during the study and within 90 days after the last dose, and should always conduct strict birth control in accordance with the label of drug/appliance and the investigator's instructions. Effective contraception measure is defined as:
        i. The sexual partner with removed deferens is the only sexual partner of female patient;
        ii. Use of any intrauterine device with documented failure rate less than 1% per year;
        iii. Double contraception, such as spermicide plus male condom, female condom, diaphragm, cervical cap or intrauterine contraceptive device.
12) Male patients should have undergone vasectomy, or agree to take effective contraception measures during the study and within 90 days after the last dose;
13) Patients are able to follow the study procedures, restrictions and requirements at the investigator's discretion.

Exclusion criteria:

1) Patients are still within 5 half-life of previous anticancer chemotherapy, biological agents or other investigational drugs (if 5 half-life exceeds 28 days, calculated as 28 days) at the time of screening;
2) Patients received systemic radiotherapy (including whole brain radiotherapy) within 28 days before enrollment, or received small area radiotherapy (stereotactic radiotherapy of central nervous system (CNS)) within 7 days before enrollment, or have not yet recovered from the previous radiotherapy;
3) Patients have not yet recovered from the toxic effects (except alopecia) caused by previous anti-tumor treatments (> CTCAE grade 1);
4) Patients underwent major surgery or have not yet been fully recovered from pervious surgery (major surgery is defined as grade 3 or 4 surgery specified in "Management Measures for Clinical Application of Medical Technology" implemented on May 1st, 2009).
5) Patients had CNS metastasis or cancer-related epilepsy requiring clinical intervention; however, patients with CNS metastasis who received treatments, or the asymptomatic patient can be enrolled;
6) Patients with a history of allergy to 5-FU or LV;
7) Patients with active HBV or HCV infection;

8) Patients diagnosed as human immunodeficiency virus (HIV) infection, or are not willing to have HIV test;
9) Patients with clinically significant active infection;
10) Patients with previous or concurrent other malignant tumors (except effectively controlled non-melanoma skin basal cell carcinoma, breast/cervical carcinoma in situ, and other effectively controlled tumors in the past 5 years even without treatments);
11) Patients with impaired cardiac function or clinically significant heart diseases, including grade 2 or higher congestive heart failure per New York Heart Association (NYHA) classification, arrhythmia, conduction abnormalities requiring treatment, cardiomyopathy, or uncontrolled hypertension;
12) Patients with serious kidney damage requiring kidney dialysis;
13) Patients with serious liver damage, grade B or C end-stage liver diseases per Child-Pugh classification (see appendix 8);
14) Any other diseases or conditions with clinical significance that may affect the protocol compliance or patient's signature of ICF at the investigator's discretion (such as uncontrolled diabetes, etc.);
15) Patients with disease of digestive tract such as duodenal ulcer, ulcerative colitis, intestinal obstruction or other conditions that may cause alimentary tract hemorrhage or perforation as judged by the investigator, or have past medical history of gastric-intestinal perforation or intestinal fistula;
16) Patient's physical condition cause the risk of investigational drug use, or render the toxicity or AE difficult to explain at the investigator's discretion.
17) Patients who received Irinotecan therapy within 3 months prior to enrollment.
18) Patients with arteriovenous thromboembolic events within the past 6 months, including myocardial infarction, cerebral stroke, deep vein thrombosis, or pulmonary embolism, etc.
19) Patients who were sensitive to Thalidomide (in Part 3).

2 Supplementary Table S1. The rules of dose de-escalation.

Agent	Dose modifications		
	First circle	After grade 3-4 AE equivalent to DLT [a]	After grade 3-4 AE equivalent to DLT again [a]
Simmitecan	First dose	25%	50%[b]
5-FU Bolus	400mg/m^2	300mg/m^2	225mg/m^2
5-FU Infusion	2400mg/m^2	1800mg/m^2	1350mg/m^2
Thalidomide	First dose	25mg	50mg [b]

[a] The above dose modification must be followed, except for the following cases: neutrophil recovery to≥1500/mm^3, and white blood cell count recovery to ≥3500/mm^3, the dose may not be modified.
[b] Compared to the first dose

3 Supplementary Table S2. Dose modification for any TEAEs leading to the study drug.

Dose modification	Part 1				Part 2				Part 3			
	L-P 50 mg/m^2 (n=3)	L-P 80 mg/m^2 (n=7)	L-P 120 mg/m^2 (n=3)	Total (n=13)	L-P 50 mg/m^2+5-FU/LV (n=3)	L-P 65 mg/m^2+5-FU/LV (n=4)	L-P 80 mg/m^2+5-FU/LV (n=3)	Total (n=10)	L-P 65 mg/m^2+ T 50mg (n=3)	L-P-80 mg/m^2+ T 50mg (n=12)	L-P 65 mg/m^2+ T 100mg (n=3)	Total (n=18)
Reduction	0	0	0	0	0	0	1(33.3)	1(10.0)	0	0	0	0
Interruption	1(33.3)	3(42.9)	1(33.3)	5(38.5)	1(33.3)	2(50.0)	1(33.3)	4(40.0)	1(33.3)	7(58.3)	2(66.7)	10(55.6)
Discontinuation	0	0	1(33.3)	1(7.7)	0	0	0	0	1(33.3)	2(16.7)	0	3(16.7)

L-P, simmitecan;5-FU/LV, 5-fluorouracil/leucovorin; T, thalidomide.

4 Supplementary Table S3. Best tumor response.

Efficacy		Part 1 n (%)				Part 2 n (%)				Part 3 n (%)			
		L-P 50 mg/m^2 (n=3)	L-P 80 mg/m^2 (n=7)	L-P 120 mg/m^2 (n=3)	Total (n=13)	L-P 50 mg/m^2+5-FU/LV (n=3)	L-P 65 mg/m^2+5-FU/LV (n=4)	L-P 80 mg/m^2+5-FU/LV (n=3)	Total (n=10)	L-P 65 mg/m^2+ T 50mg (n=3)	L-P-80 mg/m^2+ T 50mg (n=12)	L-P 65 mg/m^2+ T 100mg (n=3)	Total (n=18)
	CR	0	0	0	0	0	0	0	0	0	0	0	0
	PR	0	0	0	0	0	0	0	0	0	1(8.3)	0	1(5.6)
BOR	SD	1(33.3)	3(42.9)	2(66.7)	6(46.2)	3(100.0)	4(100.0)	1(33.3)	8(80.0)	2(66.7)	8(66.7)	0	10(55.6)
	PD	2(66.7)	4(57.1)	0	6(46.2)	0	0	2(66.7)	2(20.0)	0	2(16.7)	3(100.0)	5(27.8)
	NE	0	0	1(33.3)	1(7.7)	0	0	0	0	1(33.3)	1(8.3)	0	2(11.1)
DCR		1(33.3)	3(42.9)	2(66.7)	6(46.2)	3(100.0)	4(100.0)	1(33.3)	8(80.0)	2(100.0)	9(75.0)	0	11(61.1)
ORR		0	0	0	0	0	0	0	0	0	1(8.3)	0	1(5.6)

BOR, best overall response; CR, complete response, PR, partial response; SD, stable disease; DCR, disease control response; NE, not evaluable; ORR, objective response rate； L-P, simmitecan;5-FU/LV, 5-fluorouracil/leucovorin; T, thalidomide.
